# Association of Maternal Risk Factors with the Prevalence of Caesarean Section Deliveries: A Cross-Sectional Study

**DOI:** 10.3390/medsci11040066

**Published:** 2023-10-09

**Authors:** Eleni Pavlidou, Georgios Antasouras, Sousana K. Papadopoulou, Olga Alexatou, Dimitrios Papandreou, Maria Mentzelou, Gerasimos Tsourouflis, Aikaterini Louka, Nikolaos E. Rodopaios, Maria Chrysafi, Anastasia Sampani, Constantinos Giaginis

**Affiliations:** 1 Department of Food Science and Nutrition, University of the Aegean, 81400 Lemnos, Greece; elen.p.pavl@gmail.com (E.P.); g.antasouras@gmail.com (G.A.); rd.olga.alexatou@gmail.com (O.A.); maria.mentzelou@hotmail.com (M.M.); loukathy612@gmail.com (A.L.); m.chrisafi3@gmail.com (M.C.); 2 Department of Nutritional Sciences and Dietetics, Faculty of Health Sciences, International Hellenic University, 57001 Thessaloniki, Greece; souzpapa@gmail.com; 3 Department of Clinical Nutrition & Dietetics, College of Health Sciences, University of Sharjah, Sharjah P.O. Box 27272, United Arab Emirates; dpapandreou@sharjah.ac.ae; 4 Second Department of Propedeutic Surgery, Medical School, University of Athens, 11527 Athens, Greece; gtsourouflis@med.uoa.gr; 5 Department of Social Medicine, Preventive Medicine and Nutrition, Medical School, University of Crete, 70013 Heraklion, Greece; nikow1966@yahoo.gr; 6 First Department of Pathology, Medical School, University of Athens, 11527 Athens, Greece; gerasimos.ts@gmail.com

**Keywords:** caesarean section, pre-pregnancy, overweight, obesity, gestational weight gain, preterm birth, pregnancy complications, nutritional interventions, maternal risk factors

## Abstract

In the last few years, there has been a gradually increasing rate of caesarean section deliveries worldwide that negatively affects both mothers’ and children’s health. The present survey intended to explore the relations of common maternal risk factors with the prevalence of caesarean sections. This is a cross-sectional study including 5182 healthy mothers from geographically diverse regions of Greece, which has applied relevant inclusion and exclusion criteria. An elevated 56.4% incidence of caesarean sections was noted. The prevalence of caesarean section deliveries was estimated to be 51.5% in private hospitals and 48.5% in public hospitals. Maternal age, pre-pregnancy overweight/obesity, excess gestational weight gain, preterm birth, financial status, smoking habits, and private type of birth hospital were considerably associated with a high probability of caesarean section, regardless of several confounders. In conclusion, caesarean section rates are constantly increasing, and various maternal risk factors additively elevate its incidence, which additionally enhances the likelihood of postpartum complications for both the mothers and their infants. Public health procedures and approaches are strongly recommended to notify future mothers of the potential risk factors that may result in adverse pregnancy outcomes of caesarean section delivery, highlighting its use only for emergency medical reasons and also promoting healthier nutritional and lifestyle habits that may reduce the increasing prevalence of caesarean section deliveries.

## 1. Introduction

Caesarean sections constitute an important medical process for protecting mothers’ and children’s lives in emergency obstetric conditions, contributing to reduction of their morbidity and mortality [1]. The World Health Organization (WHO) has suggested a caesarean section rate ranging from 10% to 15% of all deliveries [2]. Nevertheless, during the previous 20 years, an exponential rise in caesarean sections has been noted worldwide [3]. The above constitutes an important public health issue since while a caesarean section may be lifesaving, it can also result in pregnancy complications for both the mothers’ and their infants’ health [1,2,3].

In our country, this issue is becoming almost similar, since more than 50% of childbirths are undergone by caesarean sections, rendering our country among the countries with the highest caesarean sections frequency worldwide. Alarmingly enough, caesarean sections’ prevalence in Greece elevated by approximately 50% during the period 1983–1996 [4]. Notably, a substantial survey by Mossialos et al. has documented a 41.6% prevalence of caesarean sections in two public health care units and a 53% incidence of caesarean sections in a private health care unit in Greece [5]. Based on these results, these authors have supported that obstetricians could decide to accomplish a caesarean section for economic and convenience purposes [5,6]. Characteristically, a large-scale, population-based study has clearly indicated that the rate of caesarean sections may continuously and more quickly increase for women delivering in private hospitals than women giving birth in public hospitals [7].

It should be emphasized that WHO has strongly supported that the population’s caesarean section rates being higher than 10% is not related to a decrease in mothers’ and their matched children’s morbidity and mortality rates, while caesarean sections can ideally be applied merely when there are strong medical recommendations [8]. Reasonably, caesarean section, similarly to other surgical processes, has been related with several short- and long-term adverse pregnancy outcomes like greater risk of uterine rupture, ectopic pregnancy, fetus mortality, preterm birth, and psychiatric symptoms [9,10]. Importantly, there are several short-term risks of caesarean sections for neonates such as diminished immune system development, greater probability of allergy, atopy, asthma [9], and severe pulmonary and body weight complications [11,12].

In the last few years, several epidemiological studies have supported that the rising rates of caesarean sections could be related with specific mothers’ risk factors. In support of this view, maternal excessive body weight prior to gestation has been considered as a significant risk factor of increasing the possibility of undergoing caesarean delivery, even if some studies were characterized by certain limitations [13,14]. Moreover, mothers’ age lower than 30 years was shown to decrease the risk of comorbidities related with caesarean section, whereas older mothers’ age, i.e., 35 years or above, has been shown to considerably enhance the rates of caesarean sections and their complications [15]. Several previous studies have also indicated that the type of residence, mothers’ educational status, financial level, and parity may considerably increase caesarean section rates; however, there are also studies that did not confirm these associations, highlighting the fact that the present results remain inconclusive [16].

Furthermore, excess gestational weight gain (GWG) higher than the Institute of Medicine (IOM) guidelines has been linked to elevated rates for caesarean section and newborns’ macrosomia [17]. There are also certain studies supporting that an increased caesarean sections’ rate might be a risk factor of preterm birth, but other studies did not confirm this finding [18,19], reinforcing the need for further investigation. Women developing gestational diabetes also have a greater likelihood of gestational hypertension and pre-eclampsia and delivering by caesarean section [20], which further leads postnatally to increasing rates of type 2 diabetes and hypertension-related disorders [21]. Much evidence has also shown that mothers who deliver by caesarean section do not frequently begin breastfeeding, or they postpone breastfeeding initiation, which could exert superior beneficial effects in both the mothers’ and their infants’ health [22]. Moreover, in parallel with the gradually increasing prevalence of caesarean sections worldwide, there is also a considerable rise in planned caesarean sections often without a clear medical reason, which may lead to rising rates of iatrogenic premature birth, and, in turn, potential adverse childhood outcomes at the next stages of their life [23]. In this aspect, the purpose of the current cross-sectional survey is to assess the possible associations of several mothers’ socio-demographic, anthropometric, lifestyle, and perinatal factors with the rates of caesarean section in a large-scale and representative population in our country by adjusting for several potential confounders.

## 2. Methods

### 2.1. Study Population

In the present study, 7191 women were initially enrolled from 11 geographically different areas of our country, rural and urban (Athens, Thessaloniki, Larissa, Kavala, Alexandroupolis, Ioannina, Patra, Kalamata, Crete, South and North Aegean). The inclusion reasons for the initial enrollment were women with a singleton childbirth during a period of 2–5 years before assignment, regardless of parity, and who had any other gestation during the above period of 2–5 years. In multiparous mothers, only the last gestation was taken into consideration. The assignment to the current study was from May 2016 to September 2020. The mothers were assigned during their visits in their personal private gynecologist or during their visits in healthcare units, e.g., public, and private hospitals.

**Figure 1:**
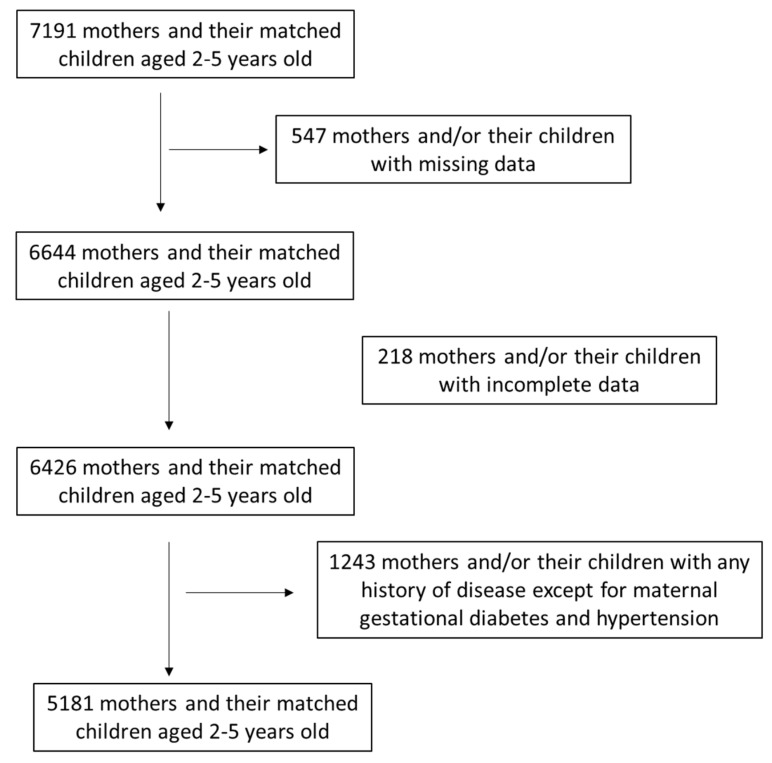
Flow chart diagram of survey assignment.

All mothers’ data were confidential. All mothers received detailed information concerning the purpose of the survey and signed a consent form giving their approval that their individual information could be announced anonymously. Sample size estimation was performed utilizing PS: Power and Sample Size calculator software, while the randomization was performed utilizing a sequence of random binary numbers (i.e., 001110110 in which 0 showed assignment and 1 not assignment to the survey). Among 7191 primarily assigned mothers, 766 mothers (10.7%) were excluded due to missing or incomplete data. Of the remaining 6425 mothers, 1243 (19.4%) of them were then excluded because of any history of disease except for maternal gestational diabetes and pregnancy-induced hypertension. Finally, 5182 healthy women were assigned in the present analysis, resulting in a final response rate equal to 72.1%. This survey was approved by the Ethics Agency of the University of the Aegean (ethics approval protocol: No. 12/14.5.2016, approval date: 14 May 2016) based on the guidelines of the World Health Organization (52nd WMA General Assembly, Edinburgh, Scotland, 2000). A flow chart diagram of the survey assignment is presented in Figure 1. 

### 2.2. Study Design

During the period of the survey, i.e., 2–5 years after delivery, relevant, validated semi-quantitative questionnaires were applied for evaluating various sociodemographic and lifestyle factors of the enrolled mothers [22,24]. Recruitment to the study was performed 2–5 years after the delivery. Mothers’ body weight at the first weeks of gestation and directly before delivery were retrieved from their individual gynecologists’ or hospitals’ medical records. These medical records included determined body weight and height information. GWG was determined by subtracting the retrieved measured body weight of the first weeks of pregnancy from the retrieved measured body weight directly before delivery. During the period of the survey, mothers’ body weight and height were also determined by qualified nutritionists, dietitians, or physicians as per protocol, i.e., 2–5 years prior to childbirth [22,24]. Body weight was determined utilizing the same electronic scale, and height was assessed utilizing a portable stadiometer [22]. The WHO recommendations were used to categorize the enrolled women as normal weight, overweight, or obese based on Body Mass Index (BMI) prior to pregnancy [24].

Maternal age, nationality, smoking habits, education status, financial level, and nulliparity/multiparity have been retrieved by relevant questionnaires 2–5 years postpartum according to mothers’ memory recall. All smoker mothers ceased smoking during pregnancy. Only multiparous women that did not have a previous caesarean section were included in the analysis. One-to-one interviews between each assigned mother and qualified scientific staff was applied to minimize recall biases. Face-to-face interviews were performed 2–5 years after deliveries during mothers’ visits to their personal gynecologists or dietitians or nutritionists or health care units. Education status was determined based on the sum of educational years and financial level was categorized based on the yearly family income as: EUR 0 ≤ 5000, EUR 1 ≤ 10,000, EUR 2 ≤ 15,000, EUR 3 ≤ 20,000, EUR 4 ≤ 25,000 and EUR 5 > 25,000. Economic level was then categorized as low for yearly income ≤ EUR 10,000, medium for yearly income > EUR 10,000 and ≤ EUR 20,000, and high for yearly income >EUR 20,000. Moreover, the type of delivery (vaginal or caesarean section) and maternal prenatal outcomes (gestational diabetes and pregnancy-induced hypertension) were also recovered by their medical files. All mothers were examined for GDM utilizing a conventional oral glucose tolerance test (OGTT) during gestation [24]. More to the point, a fasting OGTT next to 75 g glucose with a cut-off plasma glucose of ≥140 mg/dL next to 2 h for the initial and following trimester at 24–28 weeks of pregnancy had been applied for the enrolled women [24]. The cohort was additionally categorized based on the kind of hospital where the women gave birth, in public or private hospitals. Among the women who delivered by caesarean section, the type of delivery was additionally categorized as elective or emergency caesarean delivery. Emergency caesarean deliveries were performed when: (a) there were concerns for the safety of the baby, (b) there was a life-threatening emergency for the mother or her baby, (c) the mother’s labor was not progressing normally, or (d) there were complications, such as severe bleeding or severe preeclampsia.

Pregnancy-induced hypertension data were retrieved from the mothers’ medical records. Blood pressure was determined from the left arm (mmHg) by mercury sphygmomanometer. Three readings were performed on different days with the enrolled women seated, after resting for a period of 15 min in a relaxed environment and with an empty bladder, and the average of the three measurements was assessed [25]. The WHO standards were utilized by the qualified personnel concerning the diagnosis of pregnancy-induced hypertension [26]. In the current study, pregnancy-induced hypertension was classified as systolic blood pressure > 140 mmHg and diastolic blood pressure > 90 mmHg next to 20 weeks of pregnancy [27]. Only pregnancy-induced hypertension cases were included in the study because we did not have access to data concerning other pregnancy hypertensive disorders such as preeclampsia.

Furthermore, mothers were questioned if they had adopted exclusive breastfeeding for at least 4 months. To reduced recall biases, the mothers answered whether they had exclusively breastfed for at least 4 months as at the end of the 4th month and at the beginning of the 5th month, most of the mothers were counselled to progressively insert pulp foods to the nourishing habits of their infants and they can remember this time point more precisely, rendering their answers more reliable [22]. In contrast, mothers who applied breastfeeding for shorter intervals were not able to respond with high confidence concerning the accurate lactation period [22].

The enrolled women were asked to state if they had a preterm birth (<37th week) and their responses were additionally cross-checked with their medical files. Nevertheless, we detected that some of the data concerning the exact week of childbirth in medical records were not in accordance with the mothers’ answers. So, we based on medical records, and preterm birth was treated as a binary outcome as prior to and directly next to the 37th week of gestation. Mothers’ history for gestational diabetes and gestational hypertension were also recovered by their medical documents.

Detailed explaining directions were thoroughly given to the participants by the registered nutritionists, dietitians, and physicians regarding each question of the questionnaires, and a detailed presentation of the questions to enable reliable answers was rigorously accomplished.

### 2.3. Statistical Analysis

Statistical analysis was accomplished by Student’s *t*-test and one-way ANOVA for continuous variables, which were tested to have normal distribution. Kolmogorov–Smirnov test was used to assess if the continuous variables are normally distributed. Chi-square test was utilized for categorical variables. The quantitative variables which have followed normal distribution were stated as mean value ± standard deviation (SD), and the qualitative variables were stated as absolute or relative frequencies. Multivariate binary logistic regression analysis was used for evaluating if the type of delivery (caesarean section vs. vaginal) is independently related with mothers’ sociodemographic, anthropometric and lifestyle factors and maternal perinatal outcomes after adjustment for several potential confounding factors. Differences were identified as significant at *p* < 0.05 and 95% confidence interval. The statistical analysis of the study data was implemented using Statistica 10.0 software, Europe (Informer Technologies, Inc., Hamburg, Germany).

## 3. Results

### 3.1. Descriptive Statistics of the Study Population

The current survey finally assigned 5182 healthy, reproductive-aged mothers with a mean age of 37.5 ± 4.8 years. Concerning their nationality, 95.7% of the mothers were Greek and the remaining 4.3% were of other nationalities (Russian, Albanian, Ukrainian, Bulgarian, Romanian). Concerning education status of the enrolled women, the mean educational years were 14.5 ± 2.8 years (range: 6–18 years). As far as the economic status was concerned, 46.2% of the enrolled mothers stated low annual income, 45.1% medium, and 8.7% high annual income. Additionally, 25.6% of the enrolled mothers were regular smokers before gestation.

Caesarean section was applied in 56.4% of the mothers and vaginal delivery in the remaining 43.6%. Concerning the type of hospital where the deliveries were undergone, 51.5% of them were completed in private hospitals and 48.5% in public hospitals. Among caesarean sections, 47.5% were elective and 52.5% were emergency caesarean section deliveries. The mean BMI of the enrolled mothers before pregnancy was 22.7 ± 3.7 kg/m^2^ (range: 15.9–37.6 kg/m^2^). A total of 17.5% of the assigned mothers before gestation were affected by overweight, and 5.0% were affected by obesity based on their BMI status, and overall, an incidence of 22.5% overweight/obesity was noted before gestation. The mean GWG was estimated to be 13.8 ± 6.1 kg (range 4.0–45.0 kg).

Almost half of the women (49.7%) did breastfeed exclusively for at least 4 months (mean period: 4.8 ± 1.9 months), and 50.3% of the enrolled mothers did not adopt exclusive breastfeeding for at least 4 months or did not adopt breastfeeding at all. An incidence of preterm birth (<37th week) estimated at18.2% of the enrolled mothers was noted. Regarding parity, 64.2% of the enrolled mothers stated that they were nulliparous and 35.8% were multiparous. Also, 4.3% of the assigned women were diagnosed with gestational diabetes mellitus for the period of gestation, and 4.1% of the assigned women developed gestational hypertension.

### 3.2. Associations of the Mode of Delivery with Sociodemographic, Anthropometric, and Lifestyle Characteristics of the Enrolled Mothers

**Figure 2:**
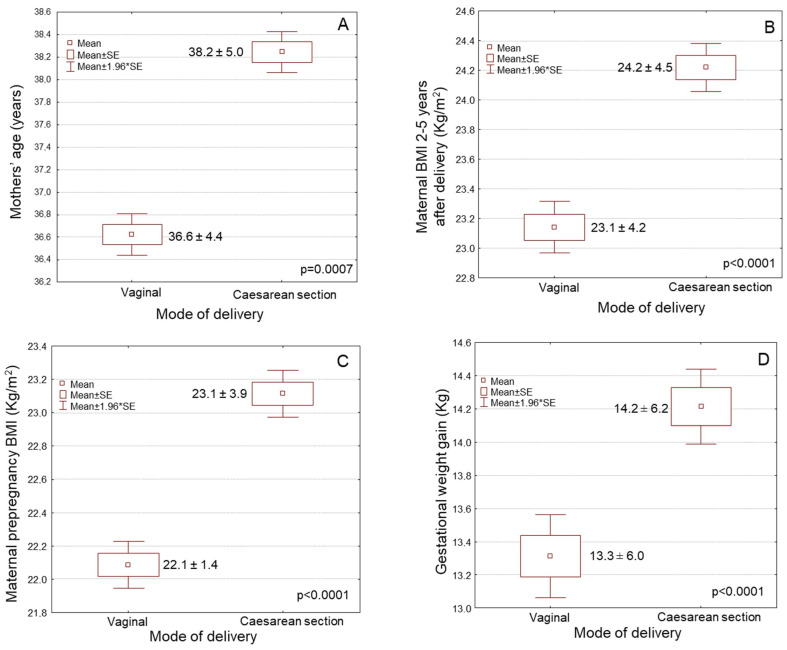
Associations of the type of delivery with: (**A**) maternal age, (**B**) maternal pre-pregnancy BMI, (**C**) maternal educational level, and (**D**) gestational weight gain.

Maternal age was significantly higher amongst the women having a child by caesarean section than those delivered vaginally (Figure 2A, Table 1, 38.2 ± 5.0 vs. 36.6 ± 4.4 years old, *p* = 0.0001). Women performing caesarean section had considerable elevated pre-pregnancy BMI values compared to those who delivered vaginally (Figure 2B, Table 1, 23.1 ± 3.8 vs. 22.1 ± 3.4 kg/m^2^, *p* = 0.0001). In crosstabulation, women performing caesarean sections were characterized by a considerably greater incidence of overweight/obesity before gestation than those delivering vaginally (Table 1, *p* ˂ 0.0001). Women delivered by caesarean section were regular smokers considerably more often compared to women giving a birth by vaginal delivery (Table 1, *p* = 0.0004). Women with elevated educational level gave a birth by caesarean section considerably more often compared to women with reduced education status (Figure 2C, Table 1, *p* = 0.0011). Women with greater economic status considerably more frequently underwent caesarean section than those of lower economic status (Table 1, *p* = 0.0117). Caesarean section was marginally but not significantly more frequently undergone in multiparous women than nulliparous women (Table 1, *p* = 0.0638). Women performing caesarean section had a significantly lower prevalence for exclusive breastfeeding compared to those performing vaginal delivery (Table 1, *p* ˂ 0.0001). No considerable difference was not found among Greek women and those of other ethnicities concerning the type of delivery (Table 1, *p* > 0.05).

### 3.3. Associations of Type of Delivery with Maternal Perinatal Factors

Women who underwent caesarean section had significantly higher GWG compared to women delivering vaginally (Figure 2D, Table 1, *p* ˂ 0.0001). Women who underwent caesarean section exhibited a significantly greater prevalence of preterm birth compared to women delivering vaginally (Table 1, *p* ˂ 0.0001). The prevalence of the caesarean sections was considerably elevated in private hospitals compared to public hospitals (Table 1, *p* = 0.0001). Private hospitals were related with a considerably greater prevalence of elective caesarean sections (56.1%) than emergency ones (43.9%), whereas public hospitals were related with a considerably greater prevalence of emergency caesarean sections (63.4%) than planned ones (36.6%) (*p* = 0.0001). Non-significant associations between mode of delivery and gestational diabetes and hypertension were noted (Table 1, *p* > 0.05).

### 3.4. Multivariate Binary Logistic Regression Analysis for Mode of Delivery by Adjustment for Multiple Confounding Factors

In the multivariate binary logistic regression analysis, the type of childbirth was related at an independent manner with mothers’ age, BMI before gestation, financial level, smoking habits, GWG, preterm birth, exclusive breastfeeding, and type of hospital delivery by adjusting for several confounders (Table 2). Women of older age (≥37.5 years) had a 58% higher probability of undergoing caesarean section compared to those of younger age (˂37.5 years) (Table 2, *p* = 0.0048). Women affected by overweight and obesity before gestation had a two-fold greater risk of undergoing caesarean section than underweight and normal weight women (Table 2, *p* = 0.0008). Women of high economic status had a 31% greater risk of delivering by caesarean section than those of low or medium economic level (Table 2, *p* = 0.0387). Regular smoker women showed a 72% greater probability of undergoing caesarean section than non-smokers (Table 2, *p* = 0.0412).

Women with higher rates of GWG (≥13.8 kg) had a 26% increased risk of delivering by caesarean section than those with lower rates (˂13.8 kg) (Table 2, *p* = 0.0094). An 84% higher likelihood of preterm birth was recorded in women undergoing caesarean section compared to women delivering vaginally (Table 2, *p* = 0.0012). Women giving birth with caesarean section exhibited a 56% greater risk of not breastfeeding exclusively for at least 4 months compared to women giving birth by vaginal delivery (Table 2, *p* = 0.0002). Caesarean sections had a two-fold higher probability to be undergone in a private hospital than in a public hospital (Table 2, *p* = 0.0021). Mothers’ ethnicity, educational status, gestational diabetes, and gestational hypertension were not related to the mode of delivery in multivariate analysis (Table 2, *p* > 0.05).

## 4. Discussion

In the last two decades, there has been a continuous rise in the prevalence of caesarean sections worldwide with considerable public attention and argument, regarding both the reasons and the adverse effects of this rise as the causes for this increase remain not fully understood [1,2,3]. More to the point, caesarean section rates have gradually risen to 19% worldwide with no notable difference on mothers and newborns morbidity and mortality, while in the southeastern European countries, they have risen to or above 50% of total deliveries [28,29]. In Greece, in 2005, a prevalence of 41.6% of caesarean sections in two public hospitals and an even higher incidence of 53% in a private hospital were previously noted; however, larger studies are recommended to confirm these rising rates [5].

In agreement with the above increasing trends, the current study revealed a 56.4% caesarean section rate during the period 2016–2020, in a nationally representative sample from 11 geographically different regions—rural, urban, and islands—in Greece. Moreover, we found that private hospitals had a slightly higher prevalence of caesarean sections than public hospitals and that the increasing rates of planned caesarean sections were slightly more frequent in private compared to public hospitals. The above association has sufficiently been established in private hospitals where planned caesarean sections were slightly more frequent than emergency ones. In this aspect, a systematic review of 17 studies suggested that several individual and social maternal reasons, involving fear of pain during delivery and apparent inequality and inadequacy of healthcare, resulted in the elevated prevalence of caesarean sections’ delivery due to mothers’ desires [30]. In accordance with our study, a greater incidence of caesarean section deliveries in private compared to public hospitals has been documented in several previous studies [31,32]. These growing caesarean section rates in private health care centers may be ascribed to the economic benefits of caesarean delivery for private institutions because of decreased birthing time, the subsequent capability of the hospital to attend more deliveries, and the higher cost of caesarean delivery [31,32]. This fact may also explain the greater prevalence of planned caesarean sections in private hospitals than public hospitals. In view of the above, the recorded significant relationship of increased rates of caesarean sections with better economic status found in the present study seems not to be exclusively random.

There is currently quite a lot of evidence that caesarean sections have been related with several mothers’ risk factors. Nevertheless, some of the available findings seem inconsistent and inconclusive, providing rather contradictory results at least for certain maternal risk factors. Currently, there is substantial evidence that older mothers’ age, i.e., 35 years or above, considerably increases the prevalence of caesarean sections and their adverse pregnancy outcomes [15,33]. In line with the above findings, we found a significantly higher age in women undergoing caesarean section than those giving a birth by vaginal delivery, regardless of several confounders that are also linked with raised probability of caesarean section delivery. In this aspect, it should be noted that as the mother’s age increases, considerably over 35 years, the probability of prenatal and perinatal complications raises [34]. Usually, amongst pregnant mothers over 35 years, the most common pregnancy complications comprise of gestational diabetes, preeclampsia, placenta previa, placental abruption, preterm delivery, low childbirth weight, small-for-gestation-age infants, fetal distress, intrauterine fetal death, gestational hypertension, and elevated perinatal morbidity and mortality [35].

Moreover, several surveys in both medium- and high-income countries reported that women of greater socioeconomic status postpone childbearing until the age of 30 years or above to carry out their education, obtain a job, and become economically secure before being pregnant [36,37]. A number of studies also documented that residency, women’s education, financial status, and parity may be considerably related with caesarean section rates [16]. In support of this view, we found that women performing caesarean section had elevated education status and greater financial level compared to women delivering vaginally; however, these associations were attenuated after adjusting for confounding factors. Multiparity also showed a marginal trend of correlation with caesarean section, at a non-significant level though. In this aspect, a recent descriptive cross-sectional study was conducted among multiparous women in a tertiary care center and showed that the prevalence of primary caesarean section in multiparous women was found to be higher than the other studies performed in similar settings [38]. Another study conducted on 8732 deliveries showed that caesarean section rates in the primiparous women were higher in all age groups when compared with multiparous women [39]. Thus, the currently available results in this topic still remain inconclusive.

There is strong evidence that suggests that mothers’ overweight and obesity before gestation increases the probability of caesarean delivery [14,37,40]. In addition, overweight and obese women who had a caesarean section experienced more adverse pregnancy outcomes than mothers with a normal body weight performing a caesarean section or women affected by obesity giving a birth by vaginal delivery [41]. In this aspect, the recommended approaches to decrease caesarean section have included strategies to reduce maternal overweight and obesity [17]. In line with the above evidence, we found a greater prevalence of overweight and obesity before gestation in women delivering by caesarean section compared to women performing vaginal deliveries, regardless of several confounders.

Furthermore, previous surveys support evidence that excess GWG above the Institute of Medicine (IOM) guidelines may increase the rates of caesarean section delivery, and the above trends are expected to elevate since the incidence of excessive body weight is constantly rising [17]. In line with the existing knowledge, the present study demonstrated that women performing caesarean sections had significantly higher rates of GWG than those delivering vaginally. In this aspect, it should be noted that only some women are usually advised about the IOM recommendations for gestational weight gain and even fewer are informed about the significance of supporting a healthy body weight status before pregnancy [42]. This signifies a lost prospect for effective prevention of pregnancy complications, since it has been reported that only 12% of women were advised correctly by health care providers, which suggests an urgent demand for improved women’s education [43].

Urgently, caesarean section has been considered as a possible risk factor of preterm birth in some previous studies [18,19,42]. Conversely, certain surveys did not support this relation, while it remains unknown whether the relation of caesarean section with preterm birth may be characterized by causality [18,19,44]. Compared with vaginal delivery, caesarean section in the second stage of labor was related with enhanced rates of subsequent spontaneous preterm birth before the 37th week of pregnancy, and early spontaneous preterm birth before the 34th week of pregnancy [19]. Alarmingly enough, preterm birth has been considered as the main cause of mortality and morbidity in early childhood with a projected worldwide rate of 10.6% and about 15 million deliveries yearly [45]. In accordance with the above studies, our study has demonstrated a positive relation of caesarean sections’ prevalence with preterm birth, independently of multiple confounders like mothers’ age, overweight/obesity, excessive GWG, gestational diabetes, and hypertension.

Pregnant women developing gestational diabetes had a higher likelihood of delivering by caesarean section [20], which may further increase the probability of diabetes mellitus type 2 and hypertension disease in mothers at the next stages of their life [21]. Preterm birth, high newborn body weight, abortion, pulmonary distress, stillbirth, and neonatal deaths have been considered among the most common adverse outcomes of gestational diabetes [20,21]. However, in our study, we found no association between caesarean section prevalence and gestational diabetes. In this point of view, it should be noted that a significant percentage of women in our study had increased pre-pregnancy overweight and obesity and excess GWG. The above factors should be included in the total analysis of delivery type, as both pre-pregnancy BMI and GWG were related with increased risk of caesarean sections, and they may act as confounding factors ameliorating the association of gestational diabetes with caesarean section [46]. Moreover, an increase in caesarean deliveries has been associated with elevated incidence for maternal pregnancy-induced hypertension [46,47,48]. However, the present survey has shown only a marginal non-significant trend of caesarean sections’ prevalence with gestational hypertension, which may be ascribed to the low number of women developing gestational hypertension included in our study population.

Caesarean section has previously been identified as an autonomous probability factor of the incompetence to begin and maintain breastfeeding, reducing the risk of any, predominant, and exclusive breastfeeding from discharge to 6 months postpartum [49,50]. In a recent substantial scoping review, analyzing 16 demographic surveys, Sonedo et al. documented that caesarean deliveries were linked to a greater probability of postponed initiation of breastfeeding as well as early interruption of exclusive breastfeeding [51]. The above findings were further confirmed by our study, highlighting the need to reduce caesarean sections’ prevalence and give specific breastfeeding assistance directly after delivery to the mothers.

Overall, public health strategies and approaches should inform the future mothers of the potential risk factors associated, promoting healthier nutritional and lifestyle habits, which may minimize the demand for undergoing caesarean section and may direct future mothers to select caesarean section merely because of emergency medical reasons. The gynecologist should further be informed of the potential risk of mothers and their infants who undergo caesarean section in an attempt to be motivated and reinforced to apply caesarean section only for medical reasons in clinical practice. Notably, decreasing the rate of caesarean sections requires a multi-faceted strategy that contains a variety of programming and monitoring initiatives. Audits of caesarean performed as part of health programs and other creative monitoring techniques can be useful tools for assessing the standard of maternity care, particularly emergency caesarean sections [52]. A wide range of non-clinical interventions to reduce unnecessary caesarean sections, mostly in high-income settings, has been notified. Few interventions with moderate- or high-certainty evidence, mainly targeting healthcare professionals (implementation of guidelines combined with mandatory second opinion, implementation of guidelines combined with audit and feedback, physician education by local opinion leader), have been shown to safely reduce caesarean section rates [53]. There is substantial evidence that one of the primary reasons behind women’s desire to give birth through a caesarean delivery is the pathological fear associated with the labor process, known under the scientific term “tocophobia”. Based on the findings of this review, the prevalence of tocophobia ranged between 7–25% among primiparous women and 7.7–16.25% among multiparous ones. Approximately 7–18.6% of women with tocophobia asked for an elective cesarean section without any medical indication [54].

The current survey had some limitations. BMI was used to categorize assigned mothers as overweight or obese. However, to expand and confirm our findings, there is a strong demand to apply more direct approaches for evaluating body fat mass and distribution. Additionally, some possible risk factors were self-reported by the enrolled women, and therefore recall biases cannot be avoided. However, we performed face-to-face interviews between each enrolled mother and qualified staff, providing a detailed explanation of each question, which reduced the recall bias. In this context, it should be noted that face-to-face interviews were not performed at the same, specific time point but between an interval of 2–5 years postpartum. The above difference in time between delivery and interview may in part influence our results. In addition, we have no available data concerning the reasons for performing emergency caesarean section deliveries, which may further influence the interpretation of the presented results. Among the primarily assigned mothers, a significant percentage of mothers (10.7%) were excluded because of missing or incomplete data, which may also influence our findings. Moreover, in spite of a careful approach to adjust for potential confounders, we acknowledge the possibility of additional confounding factors. Although we have utilized a thorough adjustment for maternal age, nationality, educational, and economic level, smoking habits, parity, and other maternal perinatal factors, there is still the possibility that additional confounders could affect our findings. In this aspect, several other lifestyle or health-associated factors, like maternal physical activity and mental health status, should be taken into consideration as potential confounders. However, we have included several potential confounders, which has strengthened the results of our study. The study sample is adequate enough and representative since it has included women from 11 geographically different regions of our country, including rural, urban, and islands areas. Nevertheless, large-scale, population-based, epidemiologic surveys with samples from several other regions of Greece are necessary for more consistent conclusions concerning the Greek population to be established. Moreover, no definitive conclusions concerning causality are able to be obtained due to the cross-sectional design of our survey despite its nationally representative nature. Lastly, we had no available data concerning how much instrumental deliveries were applied in our study population, making it difficult to extrapolate our findings to other countries where instrumental deliveries are commonly applied. Overall, several missing data should be mentioned as a limitation, meaning that the interpretation of our results should be done with caution and highlighting the strong demand to perform further studies in this topic to draw conclusive results.

## 5. Conclusions

This is a cross-sectional survey conducted on an adequate enough population of women of reproductive age, which has supported evidence that advanced maternal age, overweight/obesity before gestation, excess GWG, higher incidence of preterm birth, greater financial level, and smoking habits were considerably related to elevated probability of caesarean sections, regardless of several confounders. The incidence of the caesarean section deliveries recorded in the present study was far above the WHO recommendations. However, the present findings should be confirmed by future large-scale, population-based clinical studies with a more representative population. Moreover, to address the excess of caesarean deliveries, future strategies, and policies for stricter diagnosis of the medical indications for caesarean delivery and for preventing incentives for caesarean delivery should be accomplished.

In addition, public policies should promote healthy nutritional interventions to effectively decrease the incidence of mothers’ pre-pregnancy overweight/obesity and the excess GWG. Such public policies may considerably decrease the probability of adverse pregnancy outcomes. In this context, nutritional intervention studies applying well-recognized healthy nutritional patterns such as the Mediterranean diet may prove to be very efficient both before and during the gestation for controlling body weight of reproductive-aged women, minimizing the risk of perinatal complications and increasing the probability of healthier newborns with physiological birth body weight, and reducing risk of development pathological states during childhood such as overweight/obesity childhood asthma, diabetes mellitus, etc.

## Figures and Tables

**Table 1:** Associations of type of delivery with maternal sociodemographic, anthropometric, and lifestyle factors and maternal perinatal outcomes.

Parameters (n = 5182)	Type of Delivery		
	Vaginal (43.6%)	Caesarean (56.4%)	*p*-Value
**Mothers’ age (mean ± SE; years),** Mean age: 37.5 ± 4.8 years	36.6 ± 4.4	38.2 ± 5.0	*p* = 0.0001
**Mothers’ ethnicity (n, %)**			*p* = 0.4735
Greek (4957, 95.7%)	2169 (95.9)	2788 (95.5)	
Others (225, 4.3%)	93 (4.1)	132 (4.5)	
**Pre-pregnancy BMI (mean ± SD; kg/m^2^)** Mean value: 22.7 ± 3.7 kg/m^2^	22.1 ± 3.4	23.1 ± 3.8	*p* = 0.0001
**Pre-pregnancy BMI status (n,** **%)**			*p* < 0.0001
Underweight (149, 2.9%)	98 (4.3)	51 (1.8)	
Normal weight (3869, 74.7%)	1751 (77.4)	2118 (72.5)	
Overweight (905, 17.4%)	352 (15.6)	553 (18.9)	
Obese (259, 4.0%)	61 (2.7)	198 (6.6)	
**Educational years (mean ± SD; years)** Mean value: 14.5 ± 2.8 years	13.5 ± 2.9	14.7 ± 2.8	*p* = 0.0011
**Financial level (n, %)**			*p* = 0.0117
Low (2.937, 46.2%)	1082 (47.8)	1315 (45.0)	
Medium (2236, 45.1%)	1011 (44.7)	1325 (45.4)	
High (449, 8.7%)	169 (7.5)	280 (9.6)	
**Smoking habits (n, %)**			*p* = 0.0004
No smokers (3857, 74.4%)	1739 (76.9)	2118 (72.5)	
Regular smokers (25.6%)	523 (23.1)	802 (275.)	
**Parity (n, %)**			*p* = 0.0638
Nulliparity (3327, 64.2%)	1484 (65.6)	1843 (63.1)	
Multiparity (1855, 35.8%)	778 (34.4)	1077 (36.9)	
**Gestational weigh gain (mean ± SD; kg)** Mean values: 13.8 ± 6.1	13.3 ± 6.0	14.2 ± 6.2	*p* < 0.0001
**Preterm birth (<37th week, n, %)**			*p* < 0.0001
No (4211, 81.3%)	2190 (96.8)	2021 (69.2)	
Yes (971, 18.7%)	72 (3.2)	899 (30.8)	
**Gestational diabetes (n, %)**			*p* = 0.7019
No (4958, 95.7%)	2167 (95.8)	2791 (95.6)	
Yes (224, 4.3%)	95 (4.2)	129 (4.4)	
**Gestational hypertension (n, %)**			*p* = 0.1426
No (4968, 95.9%)	2179 (96.3)	2789 (95.5)	
Yes (214, 4.1%)	83 (3.7)	131 (4.5)	
**Exclusive breastfeeding (n, %)**			*p* < 0.0001
No (2608, 50.3%)	872 (38.6)	1736 (59.5)	
Yes (2574, 49.7%)	1390 (61.4)	1184 (40.5)	
**Hospital type of delivery (n, %)**			*p* = 0.0001
Public hospital (2522, 48.7%)	1226 (54.2)	1296 (44.4)	
Private hospital (51.3%)	1036 (45.8)	1624 (55.6)	

**Table 2:** Multivariate binary logistic regression analysis for caesarean section.

Characteristics	Caesarean Section	
	RR * (95% CI **)	*p*-Value
**Age** (below/over mean value)	1.58 (1.25–1.91)	*p* = 0.0048
**Ethnicity** (Greek/other)	0.97 (0.34–1.67)	*p* = 0.5503
**Pre-pregnancy BMI** (underweight or normal/overweight or obese)	2.14 (1.91–2.40)	*p* = 0.0008
**Educational status** (below/over mean value)	1.43 (0.88–1.99)	*p* = 0.1705
**Financial status** (low or medium/high)	1.31 (0.92–1.76)	*p* = 0.0387
**Smoking habits** (No/Yes)	1.72 (1.28–2.38)	*p* = 0.0412
**Parity** (Nulliparity/Multiparity)	1.20 (0.83–1.73)	*p* = 0.1433
**Gestational weight gain** (below/over mean value)	1.26 (0.96–1.62)	*p* = 0.0094
**Preterm birth** (No/Yes)	1.84 (1.59–2.09)	*p* = 0.0012
**Gestational diabetes** (No/Yes)	1.07 (0.23–2.04)	*p* = 0.8498
**Gestational hypertension** (No/Yes)	1.18 (0.69–1.85)	*p* = 0.2085
**Exclusive breastfeeding** (No/Yes)	0.44 (0.19–0.68)	*p* = 0.0002
**Type of hospital of delivery** (public/private)	2.05 (1.78–2.41)	*p* = 0.0021

* Relative Risk: RR; ** CI: Confidence Interval.

## Data Availability

Data are available upon request by the corresponding author.
